# Non-fatal suicidal behaviour, depression and poverty among young men living in low-resource communities in South Africa

**DOI:** 10.1186/s12889-018-6104-3

**Published:** 2018-10-22

**Authors:** J. Bantjes, M. Tomlinson, R. E. Weiss, P. K. Yen, D. Goldstone, J. Stewart, T. Qondela, S. Rabie, M.-J. Rotheram-Borus

**Affiliations:** 1 Department of Psychology, Stellenbosch University, PO Box X1, Matieland, 7602 South Africa; 2 Department of Biostatistics, University of California Los Angeles, Fielding School of Public Health, Los Angeles, CA USA; 3 Department of Psychiatry and Biobehavioral Sciences, Semel Institute, University of California Los Angeles, Los Angeles, CA USA

**Keywords:** Non-fatal suicidal behaviour, Poverty, Depression, Men, South Africa, Public health, Suicide prevention

## Abstract

**Background:**

Suicide is a serious public health problem in low- and middle-income countries. Understanding the context- and gender-specific risk factors for non-fatal suicidal behaviour is the cornerstone of evidence-based public health interventions to reduce suicide. Poverty and symptoms of depression are well established risk factors for suicidal behaviour. However, little is understood about how proximal economic factors (such as losing one’s job, or food insecurity) may confound the effects of symptoms of depression to increase the risk of non-fatal suicidal behaviour in vulnerable populations, such as young men living under conditions of endemic poverty. The aim of this study was to explore the extent to which a wide range of poverty-related variables account for non-fatal suicidal behaviour independent of, or in addition to, symptoms of depression among young men living in low-resource communities in South Africa (SA).

**Methods:**

Data were collected from a clustered sample of 647 young men living in low-resource communities in the Western Cape province of SA. Multivariate regressions were used to identify the associations between poverty-related measures, symptoms of depression, and past-month prevalence of non-fatal suicidal behaviour.

**Results:**

Non-fatal suicidal behaviour in the last month was reported by 47 (6.13%) participants: suicidal ideation (*n* = 43; 5.97%); suicide plan (*n* = 5; 0.77%); suicide attempt (*n* = 4; 0.62%), and deliberate self-harm without intent to die (*n* = 4; 0.62%). Past-month prevalence of non-fatal suicidal behaviour was significantly associated with particular dimensions of poverty (living in a home without a toilet on the premises, having previously been fired, and food insecurity), but not with other dimensions of poverty (such as prolonged unemployment and low levels of income). However, symptoms of depression were a more significant predictor of non-fatal suicidal behaviour than any measure of poverty (aOR=1.093, 95% CI=1.058-1.129, *p* < .000).

**Conclusions:**

Depressive symptoms are more strongly associated with non-fatal suicidal behaviour than a range of proximal and distal economic factors among young men living under conditions of endemic poverty in South Africa. This has important public health implications and highlights the importance of increasing young men’s access to psychiatric services and targeting depression as an integral component of suicide prevention in low resource communities.

**Electronic supplementary material:**

The online version of this article (10.1186/s12889-018-6104-3) contains supplementary material, which is available to authorized users.

## Background

Suicidal behaviour is a global public health problem [1]. Approximately 75% of suicides occur in low- and middle-income countries (LMICs), yet the majority of what is known about suicidal behaviour comes from high-income, Western countries. Understanding the context- and gender-specific risk factors for non-fatal suicidal behaviour is the cornerstone of evidence-based public health interventions to reduce suicide, given that non-fatal suicidal behaviour is associated with increased risk of suicide and given that there are significant gender differences in the aetiology of suicide and variations in patterns of suicidal behaviour across different geographic regions of the world [1]. Additionally, it is important to identify proximal risk factors for suicidal behaviour among groups at high risk of suicide; suicide prevention interventions targeting proximal risk factors (such as depression or unemployment) may be more efficient and effective to implement than strategies which seek to address systemic distal risk factors (such as endemic poverty, hegemonic models of masculinity, and cultural norms about suicide) [1]. There are clearly delineated groups of individuals at elevated risk of suicide, for example gay individuals, young men, people living in poverty, homeless people, and individuals with psychiatric symptoms [2]. What is less clear in the literature is how proximal factors (such as losing one’s job, food insecurity, or experiencing symptoms of depression) may increase the risk of suicidal behaviour among those who are already at elevated risk of suicide by virtue of distal factors (such as growing up under conditions of poverty or being male) [3]. In this context we set out to investigate the extent to which a wide range of poverty-related variables account for non-fatal suicidal behaviour independent of, or in addition to, symptoms of depression among a clustered sample of 647 young men living in low-resource peri-urban communities in the Western Cape province of South Africa (SA). We studied young men living under conditions of poverty given that: (1) 80% of deaths by suicide in SA are male and the majority of suicides in the country occur among individuals between the ages of 18 and 24 [4]; and (2) poverty has consistently been associated with suicidal behaviour in LMICs [3]. We were interested in investigating how proximal factors (such as symptoms of depression, food insecurity, and job loss) might increase the risk of suicide among young men who are already at elevated risk by virtue of the fact that they live in communities where poverty is endemic. Additionally we focused on poverty-related correlates of symptoms of depression, given the extensive literature of associations between depression and suicidal behaviour [2].

### Definition of non-fatal suicidal behaviour

The term “suicidal behaviour” has been used in the World Health Organization (WHO) Suicide Report to refer to the entire spectrum of suicidal phenomena; “suicidal behaviour refers to a range of behaviours that include thinking about suicide (or ideation), planning for suicide, attempting suicide and suicide itself” (p. 12) [1]. A distinction is made between suicide deaths and non-fatal suicidal behaviour [5]. Non-fatal suicidal behaviour denotes suicidal ideation and behaviours directed towards intentionally ending one’s life but which do not result in death (i.e., deliberate self-harm). Suicidal ideation is a cognitive occurrence characterised by thoughts of death and a desire to die; suicidal ideation includes the wish or desire to die, thoughts of killing oneself without any intent to act on these, and intentions to kill oneself, including making suicide plans [6]. In this paper, we have used the term “non-fatal suicidal behaviour” to denote any suicidal behaviour with a non-fatal outcome, irrespective of whether death was intended. This broad use of the term is in keeping with the terminology used by WHO and is aligned with expert consensus that suicide prevention efforts should focus on the full spectrum of suicidal behaviour, including passive suicidal ideation (i.e. thoughts of death), active suicidal ideation (i.e. thoughts of ending one’s life) and deliberate self-harm, irrespective of intention to die [1].

Links between non-fatal suicidal behaviour and suicide deaths are contested. While some authors have reported an association between these phenomena, other authors have found no relationship between suicide and non-fatal suicidal behaviours [7, 8]. This has given rise to speculation that non-fatal suicidal behaviour and suicide are separate but overlapping phenomena, each with its own set of risk factors. Nonetheless, there is evidence that non-fatal suicidal behaviour can predict future suicide attempts [9, 10]. In adolescent and adult populations, suicidal ideation has been shown to predict both suicide attempts [11–13] and suicide [14]. Some forms of passive suicidal ideation have also been shown to predict suicide; individuals reporting a wish to die are five to six times more likely to die by suicide compared to the general population [14]. However, other forms of passive suicidal ideation, such as the belief that one would be better off dead or thoughts of one’s own death, have not been consistently associated with increased risk of suicide [5].

### Psychiatric and socio-economic correlates of suicidal behaviour

Five decades of epidemiological and risk factor research has established that suicidal behaviour is associated with psychiatric disorders, principally depressive disorders, substance use disorders, psychotic illnesses, and personality disorders [2]. There is, however, a growing body of literature, building on Durkheim’s work, which asserts that socio-cultural and economic contexts are also significant factors in the aetiology of suicidal behaviour, and that it is important to expand our understanding beyond the psychiatric determinants of this behaviour [15–17]. Critical suicidologists [18] have gone so far as to assert that “suicide is about far more than mental disorders, and may be about something quite different” (p. 1370), although it is not entirely clear what the empirical evidence is to support such claims.

This renewed focus on contextual and socio-economic factors has spurred a wave of research investigating associations between economic variables and suicidal behaviours. Suicidal behaviours have been associated with a range of poverty-related measures, including unemployment, indebtedness, economic inequalities, and economic shocks [4, 19]. There is a growing body of literature suggesting that socio-economic factors, such as poverty and living circumstances, may also constitute risk factors for suicidal behaviour [3]. However, the overwhelming majority of studies on poverty and suicidal behaviour focus on narrow measures of poverty (such as unemployment) and measure associations between poverty and suicidal behaviour without exploring the potential influence of mental illnesses (such as depression) and co-factors (such as gender and age) [20].

In order to plan suicide prevention interventions, it is necessary to understand how proximal psychiatric and economic risk factors interact with distal socio-economic and contextual factors to precipitate suicidal behaviour. A meta-analysis of 350 studies investigating risk factors for suicidal behaviour concluded that experts’ abilities to predict if someone will engage in suicidal behaviour is no better than chance [2]. Franklin et al. [2] speculate that this lack of precision is in large part a result of the fact that studies in this field have investigated risk factors in isolation and failed to take account of potential interactions between variables.

There are good reasons for investigating how a wide range of poverty-related socio-economic factors interact with psychiatric factors to precipitate suicidal behaviour, particularly in LMICs where psychiatric and mental health care resources are scarce [20]. Understanding the interaction between proximal and distal factors has implications for targeted suicide prevention interventions and for planning non-psychiatric suicide prevention interventions in low-resource communities. Many suicide prevention programmes focus on identifying at-risk individuals and promoting access to psychiatric care [21]. There are alternative suicide prevention programmes that do not rely on diversion of at-risk individuals to psychiatric care. For example, some interventions focus on screening for imminent danger and then working with high-risk individuals to identify adaptive behavioural repertoires and develop adaptive skills (such as effective communication and problem-solving), which protect against suicidal behaviour [22]. Fewer suicide prevention programmes have utilised population-based risk reduction approaches or focused explicitly on addressing structural and macro-environmental factors (such as food insecurity or unemployment).

## Methods

The aim of this cross-sectional study was to investigate the prevalence and poverty-related correlates of non-fatal suicidal behaviour among a cluster sampled group of young men living in low-resource communities in the Western Cape province of SA. We were interested in the extent to which a wide range of dimensions of poverty accounted for non-fatal suicidal behaviour independent of, or in addition to, measures of depression. Additionally we were interested in poverty-related correlates of depressive symptoms, given that suicidal behaviour is strongly associated with mood disturbances.

### Setting

Data were collected in Khayelitsha and Mfuleni, two peri-urban townships in the greater Cape Town area. Khayelitsha has a conservatively estimated population of 391,749 (as of 2011) and covers an area of approximately 43.51 square kilometres (16.80 square miles) [23]. The median average household income in this community is ZAR20,000 (approx. US$1,508 at the time of the study) per annum compared to the Cape Town City median of ZAR40,000 (US$3,016), making the township one of the poorest areas of Cape Town [24]. Approximately half of Khayelitsha’s residents live in informal housing. There are five major settlements with formal and informal housing in this township. Mfuleni is a relatively new township located close to Khayelitsha and has a population of approximately 52,300 people. Reliable estimates of family household income in Mfuleni are not available, but the living conditions in this township are considered comparable with those of Khayelitsha. Within each of these settlements we used aerial maps to identify 18 neighbourhoods matched on density, ratio of dwellings to shebeens (bars), access to day-care and health care clinics, and the availability of water and toilets on-site. Within each of the 18 neighbourhoods, there was formal and informal housing and each neighbourhood contained approximately 450-600 households.

### Sampling and recruitment

Approximately 50 young Black African men aged 18-29 years old were recruited from each of the 18 neighbourhoods. Trained recruiters went from dwelling to dwelling, randomly selecting the first household (by flipping a coin on a hardcopy of the neighbourhood map) and then systematically approaching houses in concentric circles, to identify approximately 50 young men aged 18-29 years per neighbourhood. To be included in the study the young men had to (a) have slept at least four nights per week in the household for the two months prior to recruitment, (b) be able to speak isiXhosa or English, and (c) be able to understand the recruiter. Young men meeting the inclusion criteria were invited to participate in an assessment interview conducted in a safe and confidential setting at a time convenient to them. A total of 647 young men who had been recruited agreed to participate in the study, yielding a participation rate of 72%. Men who chose not to participate were not asked to give reasons for their decision, so we are not able to report reasons for non-participation.

### Procedure

Data were collected between August 2016 and April 2017 from 647 participants by trained interviewers who administered a one-hour assessment recording participants’ responses on mobile phones using the Mobenzi data-collection platform. Participants received reimbursement of ZAR120 (approx. equal to US$9 at the time of the study) for their time.

### Data collection and measures

The following data were collected:

- *Demographic variables*: Data on participants’ age, partnership status (married, living together, casual relationship), number of children, level of completed education, and employment status were collected using a demographic questionnaire developed by Kalichman, Simbayi, Vermaak, Jooste, and Cain [25] for use in SA.
- *Income and employment*: Participants were asked about their employment status, nature of current work, monthly income, whether they were satisfied or dissatisfied with their current remuneration, income received in the past three months, highest level of income ever, financial support from parents, financial support from partner, employment before the age of 18, longest period employed, employed in the last year, ever having been fired from a job, fired from a job in the last year, and number of financial dependants. The number of financial dependents was categorised as: 0 (reference group), 1, and 2 or more. Longest job held was categorised as: never employed (reference group), employed for less than six months, and employed for six months or longer.
- *Housing and living circumstances*: Participants were asked details of recent moves, how long they had lived in their current abode, details of co-habiting (number of people and relationship to them), type of housing (formal versus informal), water source, household toilet, electricity, and type of cooking fuel used.
- *Food insecurity*: Items taken from the Household Food Insecurity Access Scale (HFIAS) were used to assess food insecurity. The HFIAS was developed as a simple means of assessing household food insecurity using a standardised questionnaire composed of nine questions which ask about the occurrence and frequency of different dimensions of food security in the past four weeks. This instrument has been used in several countries and appears to distinguish food insecure from food secure households across different cultural contexts, including SA [26, 27]. We asked participants: (1) how many days they had gone hungry in the past week, and (2) how many days a child in the family had gone hungry in the past week. We also asked them how often (never, rarely, sometimes, often) each of the following events occurred in the past four weeks as a result of lack of money: (1) worried about household’s supply of food; (2) not able to eat the kinds of foods you preferred; (3) not able to eat certain kinds of food; (4) ate the same food each day; (5) ate smaller meals than usual; (6) ate fewer meals; (7) went to sleep hungry; (8) went without food for the entire day; and (9) there was no food in the house. We analysed each of these items individually and coded the responses to the questions about the frequency of each occurrence as 0 (rarely or never) and 1 (sometimes or often). We also created a total food security score by adding the responses to each of the 9 items, to yield an aggregate measure of food insecurity ranging from 0 to 9.
- *Symptoms of depression*: The Center for Epidemiologic Studies Depression Scale (CES-D) was used to measure symptoms of depression. The scale was developed as a screening tool [28] and is one of the most widely used instruments in psychiatric epidemiology [29]. This 20-item self-report depression inventory asks participants how they felt or behaved during the past week. Scores range from 0 to 60, with higher scores indicating greater symptoms of depression. A total score of 16 or higher is considered to be a clinically significant cut-off for Major Depressive Disorder. The scale has been found to be reliable (α > .85) and has been used in SA [30, 31].
- *Non-fatal suicidal behaviour:* The Columbia-Suicide Severity Rating Scale (C-SSRS), adapted to include items measuring deliberate self-harm without intent to die, was used to assess lifetime and one-month prevalence of suicidal ideation, self-harm without intent to die, thoughts of suicide without a plan, suicide plan, and suicide attempt [10]. The C-SSRS has been used in other SA studies to investigate the prevalence of non-fatal suicidal behaviour [32]. For the purpose of statistical analysis, we dichotomised participants into two groups; those who did not report any non-fatal suicidal behaviour in the past month (reference group), and those who reported any form of non-fatal suicidal behaviour in the past month.

The questionnaires and data collection procedures were initially tested and refined in a pilot study which was conducted with a smaller sample prior to the commencement of this project. Quality checks were conducted during data collection to ensure that data collectors were following the protocol. The interview guide is included as a Additional file 1.

### Data analysis

Data were analysed with SAS 9.4. We summarised all variables and drew plots of scores for depressive symptoms and food insecurity. We used the Cochran-Mantel-Haenszel Test, to test for association between all independent variables and measures of non-fatal suicidal behaviour, after adjusting for neighbourhood. We fit univariate linear regressions of predictors to CES-D and combined significant predictors (*p* < .05) into a multivariate regression. Having a toilet on-site had a p-value of .06 but was also carried over into the multivariate regression. We fit single predictor logistic regressions to model non-fatal suicidal ideation, followed by a multivariate logistic regression without depressive symptoms. We then ran a multivariate logistic regression including symptoms of depression as a predictor of non-fatal suicidal behaviour. For both multivariate logistic regressions, we retained significant or near-significant univariate predictors (*p* < .10), and we forced the following variables into the model: age, food insecurity, and number of children.

### Ethical considerations

Permission to conduct this study was obtained from the Health Sciences Research Ethics committees at the University of Stellenbosch and the University of California, Los Angeles. Written informed consent was obtained from all participants prior to data collection. Privacy and confidentiality were protected by collecting data in a private space and storing data in such a way that participants could not be identified. De-identified data were stored and accessed via a password-protected, cloud-based database. All participants who reported any form of non-fatal suicidal behaviour in the last month were assessed for current suicidal ideation and intent, and were referred to an appropriate mental health professional, if indicated.

## Results

The sample consisted of 647 Black-African men between the ages of 18 and 29. The majority of the sample had attended high school (*n* = 618, 96%), were in romantic relationships (*n* = 576, 90%), did not report clinically significant symptoms of depression (*n* = 455, 70%), and did not report any non-fatal suicidal behaviour in the past month (*n* = 600, 93%). Most participants (*n* = 630, 97%) had low past-month income ( < ZAR5,000, US$377) and most of those who were employed were dissatisfied with their income (*n* = 443, 68%). Demographic details of the sample are presented in Table 1, with the results of the Cochran-Mantel-Haenszel test for association with non-fatal suicidal behaviour.

**Table 1:** Sample characteristics (*N* = 647 men)

	No suicidal behaviour	Non-fatal suicidal behaviour	Cochran-Mantel-Haenszel Test statistic (df)	*p*-value
	*N* = 600	*N* = 47		
Age in years, mean (sd)	23.0 (2.9)	26.2 (15.5)	-	0.17
Attended High School, Count (%)	573 (95.5)	45(95.7)	0.0154 (1)	0.90
Completed High School, Count (%)	186 (31%)	4 (9%)	9.4722 (1)	0.00*
Single	61 (10%)	10 (21%)		0.05
Number of Recognised Children, Count (%)			4.3739 (3)	0.22
0	462 (93%)	35 (7%)		
1	114 (93%)	8 (7%)		
2	20 (83%)	4 (17%)		
3	4 (100%)	0 (0%)		
Brick Housing	304 (51%)	21 (45%)	0.0823 (1)	0.77
Water Availability			3.6576 (2)	0.16
In the Home	229 (38%)	13 (28%)		
On the Premises	196 (33%)	15 (32%)		
Community Tap	172 (29%)	19 (40%)		
Toilet			9.2192 (4)	0.06
Flushing toilet on the Premises	405 (68%)	24 (51%)	2.7249 (1)	0.10
Public	142 (24%)	15 (32%)		
Portable	10 (2%)	2 (4%)		
Bucket System	9 (2%)	0 (0%)		
Bush	31 (5%)	6 (13%)		
Electricity	595 (99%)	47 (100%)	0.1789 (1)	0.67
Cooking Fuel - Electricity	516 (86%)	43 (91%)	0.7460 (1)	0.39
Ever having been employed as a:			3.2946 (2)	0.19
Builder	101 (17%)	12 (26%)		
Other Work As Defined in Questionnaire	325 (54%)	29 (62%)		
Never Worked	141 (24%)	7 (15%)	2.1607 (1)	0.14
Past Month’s Income			7.5696 (4)	0.11
0 to 499 rand	271 (45%)	30 (64%)		
Does not think this is good income	201 (74%)	24 (80%)		
500 to 1000 rand	128 (21%)	8 (17%)		
Does not think this is good income	92 (72%)	6 (75%)		
1001 to 2000 rand	90 (15%)	3 (6%)		
Does not think this is good income	55 (61%)	2 (67%)		
2001 to 5000 rand	94 (16%)	6 (13%)		
Does not think this is good income	50 (53%)	4 (67%)		
5001+ rand	17 (3%)	0 (0%)		
Does not think this is good income	9 (53%)	0		
Monthly Income 3 Months Ago			5.6121 (4)	0.23
0 to 499 rand	240 (40%)	26 (55%)		
Does not think this is good income	192 (80%)	22 (85%)		
500 to 1000 rand	154 (26%)	10 (21%)		
Does not think this is good income	107 (69%)	8 (80%)		
1001 to 2000 rand	83 (14%)	5 (11%)		
Does not think this is good income	47 (57%)	4 (80%)		
2001 to 5000 rand	103 (17%)	6 (13%)		
5001+ rand	20 (3%)	0 (0%)		
Dissatisfied with income in the past month or past 3 months	424 (71%)	39 (83%)	1.9348 (1)	0.16
Highest Income Ever, Median (IQR)	2800 (1500, 4500)	2000 (1000, 4000)		
Receives Income From Parents	127 (21%)	4 (9%)		0.05*
Receives Income from Partner			3.9737 (1)	0.41
Yes	47 (8%)	2 (5%)		
No	194 (34%)	11 (26%)		
Partner Has No Income	329 (58%)	30 (70%)		
Employed Under 18	99 (17%)	10 (21%)	0.4221 (1)	0.52
Job Longest Length			4.6188 (2)	0.10
Never Had a Job	140 (23%)	7 (15%)		
< 6 months	234 (39%)	15 (32%)		
>= 6 months	226 (38%)	25 (53%)		
Two or More Jobs in the Last Year	176 (29%)	24 (51%)	9.0176 (1)	< 0.00*
Has Been Fired More than 1 Time In Life	16 (3%)	6 (13%)	10.7869 (1)	< 0.00*
Has Been Fired in the Last Year	24 (4%)	6 (13%)	8.0487 (1)	< 0.00*
Number of People Supporting			2.3404 (2)	0.31
None	378 (63%)	24 (51%)		
One to Two	182 (30%)	18 (38%)		
Three or More	40 (7%)	5 (11%)		
Days Hungry in Past Week, Median (IQR)	1 (0, 2)	2 (1, 3)		
Hungry 1 or more days a week	312 (52%)	41 (87%)	22.2211 (1)	< 0.00*
Hungry 3 or more days a week	88 (15%)	17 (36%)	12.9910 (1)	< 0.00*
Hungry 4 or more days a week	33 (6%)	8 (17%)	9.7243 (1)	< 0.00*
Days Children Hungry in Past Week, Median (IQR)	0 (0, 0)	0 (0, 2)		
Children Hungry 1 or more days a week	131 (22%)	17 (36%)	4.8922 (1)	0.03*
Children Hungry 3 or more days a week	35 (6%)	6 (13%)	2.7915 (1)	0.10
Children Hungry 4 or more days a week	15 (3%)	2 (4%)	0.5678 (1)	0.45
Worried about Household Food Supply			14.3480 (1)	< 0.00*
Never or Rarely	368 (61%)	16 (34%)		
Sometimes or Often	232 (39%)	31 (66%)		
Not able to eat the kinds of food you prefer			6.1330 (1)	0.01*
Never or Rarely	312 (52%)	16 (34%)		
Sometimes or Often	288 (48%)	31 (66%)		
Not Able To Eat Certain Kinds of Food B/c of Money			6.1330 (1)	0.01*
Never or Rarely	312 (52%)	16 (34%)		
Sometimes or Often	288 (48%)	31 (66%)		
Same Food Each Day			14.2851 (1)	< 0.00*
Never or Rarely	341 (57%)	14 (30%)		
Sometimes or Often	259 (43%)	33 (70%)		
Smaller Meals			4.9269 (1)	0.03*
Never or Rarely	367 (61%)	21 (45%)		
Sometimes or Often	233 (39%)	26 (55%)		
Less Meals a Day			16.9928 (1)	< 0.00*
Never or Rarely	397 (66%)	17 (36%)		
Sometimes or Often	203 (34%)	30 (64%)		
Go To Sleep Hungry			16.8415 (1)	< 0.00*
Never or Rarely	523 (87%)	31 (66%)		
Sometimes or Often	77 (13%)	16 (34%)		
Whole Day Without Food			16.8602 (1)	< 0.00*
Never or Rarely	525 (88%)	31 (66%)		
Sometimes or Often	75 (13%)	16 (34%)		
No Food in House			20.2315 (1)	< 0.00*
Never or Rarely	519 (87%)	29 (62%)		
Sometimes or Often	81 (14%)	18 (38%)		
Overall Hunger Score (range 0 to 9) median (IQR)	2 (1, 5)	6 (2, 8)		*p* < 0.00*

*are statistically signficant

### Prevalence of non-fatal suicidal behaviour

Non-fatal suicidal behaviour in the last month was reported by 47 (6.13%) participants: suicidal ideation (*n* = 43; 5.97%); suicide plan (*n* = 5; 0.77%); suicide attempt (*n* = 4; 0.62%), and deliberate self-harm without intent to die (*n* = 4; 0.62%).

The lifetime prevalence of having made a suicide attempt was 6%, with 30, seven, and three individuals reporting exactly one, two, and three or more lifetime suicide attempts, respectively. Eight individuals reported their last suicide attempt to be within the previous 12 months, three between one to two years prior, and 29 more than two years prior to data collection.

### Prevalence of symptoms of depression

CES-D scores ranged from 0 to 46 for the sample of 647 young men. The median score was 9 (IQR: 4-18). A total of 192 (30%) of the sample reported CES-D scores greater than 16, indicating clinically significant symptoms of depression. The mean CES-D score in the 600 men who had not reported non-fatal suicidal behaviour in the past month was 11, while the mean CES-D score in the 47 men who had exhibited non-fatal suicidal behaviour in the last month was 25. This difference in CES-D scores between men who reported non-fatal suicidal behaviour and those who did not was highly significant (*p* < .000).

### Predictors of symptoms of depression

Table 2 reports results of symptoms of depression regressed onto all demographic and poverty-related predictor variables. In univariate analysis, increased symptoms of depression were significantly associated with: older age, receiving monthly income from parents, dissatisfaction with current income, having ever been fired, never having had a job (as compared to longest job held less than six months, or greater than or equal to six months), having financial dependants, and food insecurity.

**Table 2:** Multivariate Linear Regression with symptoms of depression as outcome measure and poverty variables as predictors

Variable	Adjusted B estimate	SE	*P*-value	Univariate *P*-value
Intercept	2.72	2.23		
Age, years	0.14	0.08	*0.064*	< 0.0001*
Has a toilet on premises	-0.76	0.74	0.30	*0.067*
Receives income from parents	0.50	0.88	0.57	0.0047*
Dissatisfied with income of past month or past 3 months	1.20	0.82	0.15	0.0015*
Has been fired before	2.51	1.01	0.013*	< 0.0001*
Food insecurity score	1.64	0.13	0.0001*	< 0.0001*
Longest job held for < 6 months (reference group: never had a job)	-1.66	0.93	*0.074*	0.066*
Longest job held for >= 6 months (reference group: never had a job)	-1.40	1.03	0.18	0.012*
Financially supports one individual (reference group: no financial dependants)	0.07	0.92	0.94	0.72
Financially supports two or more individuals (reference group: <2 financial dependants)	3.33	0.96	0.0006*	< 0.0001*

*are statistically signficant

When adjusting for all variables, increased symptoms of depression were predicted by: having previously been fired, food insecurity, and financially supporting two or more dependants.

### Predictors of non-fatal suicidal behaviour

Table 3 shows the results of logistic regression analysis with non-fatal suicidal behaviour as the outcome, and demographic and poverty-related measures as the independent variables. In the univariate analysis, non-fatal suicidal behaviour was associated with: not having attended high school, having fewer children, not receiving income from parents, having less income, and higher levels of food insecurity. In the multivariate regression, non-fatal suicidal behaviour was significantly predicted by: not having a toilet on the premises, having previously been fired, and higher food insecurity. The likelihood ratio test indicated that the logistic regression model was statistically significant as a whole, χ^2^ (9) = 48.8, *p* < .000.

**Table 3:** Logistic regression analysis with nonfatal suicidal behaviour as outcome measure and poverty variables as predictors

Variable	Adjusted Odds Ratio	95% CI	*P*-value	Univariate *P*-value
Age, years	1.060	[0.958, 1.173]	0.26	0.90
Attended high school	0.813	[0.171, 3.858]	0.79	0.031*
Single	2.167	[0.748, 6.279]	0.15	0.33
Number of recognised children	0.923	[0.524, 1.625]	0.78	0.014*
Toilet on the premises	0.479	[0.245, 0.934]	0.031*	0.055
Receives income from parents	0.597	[0.199, 1.784]	0.35	0.0001*
Has been fired before	2.646	[1.252, 5.592]	0.011*	0.088
Income	0.980	[0.951, 1.009]	0.18	0.021*
Overall food insecurity score	1.266	[1.119, 1.432]	0.0002*	< 0.0001*

*are statistically signficant

Table 4 displays logistic regression results for non-fatal suicidal behaviour, with CES-D depression scores included as an independent variable. In this analysis, all variables that had been significant in the univariate analysis and multivariate regression, were no longer significant at alpha = .05. However, the association between CES-D scores and non-fatal suicidal behaviour was highly significant. Not having a toilet on the premises and having previously been fired were almost significantly (*p* = .06) associated with increased odds of non-fatal suicidal behaviour. The likelihood ratio test indicated that the logistic regression model was statistically significant as a whole, χ^2^ (10) = 78.9, *p* < .000.

**Table 4:** Logistic regression with non-fatal suicidal behaviour including symptoms of depression

Variable	Adjusted Odds Ratio	95% CI	*P*-value
Age, years	1.038	[0.943, 1.143]	0.44
Attended high school	0.926	[0.167, 5.124]	0.93
Single	1.933	[0.610, 6.130]	0.26
Number of recognised children	1.121	[0.622, 2.019]	0.70
Toilet on the premises	0.514	[0.253, 1.043]	0.065*
Receives income from parents	0.659	[0.209, 2.076]	0.48
Has been fired before	2.100	[0.957, 4.610]	0.064*
Income	0.983	[0.955, 1.013]	0.27
Overall food insecurity score	1.099	[0.960, 1.258]	0.17
CES-D*	1.093	[1.058, 1.129]	< 0.0001*

*CES-D had *p* < .000 in the univariate regression as well

*are statistically signficant

## Discussion

The one-month and lifetime prevalence of non-fatal suicidal behaviour in our sample of young Black-African men living in low-resource communities in SA was lower than the prevalence reported for men in the general population of the country. Data collected in the South African Stress and Health Survey between 2002 and 2003, estimated lifetime prevalence of suicidal ideation, suicidal plans and suicidal attempts at 8.0, 3.3 and 1.8% respectively, in a nationally representative sample of males [33].

Thirty percent of our sample reported clinically significant symptoms of depression over the one-week period prior to assessment, as indicated by a score of greater than 16 on the CES-D. This is significantly higher than the prevalence of Major Depressive Disorder (MDD) typically found in the general population of the country. Tomlinson et al. [31], for example, reported lifetime and one-month prevalence rates for MDD of 9.7 and 4.9%, respectively. It is not immediately apparent from our data why the prevalence of clinically significant symptoms of depression would be so marked among our study population, although this may in part reflect the adverse socio-economic conditions under which these young men live and the high levels of hopelessness which accompanies their lack of economic opportunities.

In this study, symptoms of depression were significantly associated with food insecurity, having been fired, and having two or more financial dependants. Our finding that poverty-related variables and food insecurity were significantly associated with symptoms of depression in a community sample of men living in peri-urban settlements in SA, is consistent with other literature from LMICs [34]. It is significant that most participants reported low incomes, were dissatisfied with their income, and were financially responsible for two or more others. Socio-economic stressors, especially financial stress, are known to increase the likelihood of developing symptoms of depression [35, 36], which may account for the observed association between poverty and depressive symptoms, and the high rates of depressive symptoms in our sample.

Although food insecurity and job losses were associated with depressive symptoms, the causal pathway of the relationship among these variables is unknown. It is possible that depression might cause a person to be fired from their job (as a result of missing work or not fulfilling work-related requirements), but it is equally possible that being fired from one’s job might give rise to depressive symptoms [37], and both pathways might well apply to different people. Future longitudinal studies could assess the temporal relationship between measures of poverty and measures of depression to help shed light on the interaction of these variables in community samples of young men living under conditions of poverty.

We found that non-fatal suicidal behaviour was significantly associated with a range of poverty-related measures, including not having a toilet on the premises, having previously been fired, and food insecurity. Non-fatal suicidal behaviour in this sample was not, however, associated with other poverty-related variables such as availability of water, access to electricity, being unemployed, past month income, income in the past three months, satisfaction with income, receiving financial assistance from a partner or parent, longest length of employment, number of jobs in the last year, and number of financial dependants. This suggests that while poverty may indeed account for some of the variance in non-fatal suicidal behaviour, it would seem that there may be specific aspects of poverty that are important determinants of non-fatal suicidal behaviour in this sample, rather than poverty per se.

It is not clear from our data why variables such as not having a toilet on the premises, having previously been fired, and food insecurity would be associated with non-fatal suicidal behaviour. However, such experiences are typically associated with shame, loss of dignity, and hopelessness [38], which may explain why they would be associated with non-fatal suicidal behaviour. A large body of literature has shown associations between suicidal behaviour and shame [39]. It is significant that in this community, problems related to unemployment, low income, frequent changes of jobs and receiving financial assistance from a partner or parent, are endemic and may thus constitute more of a shared experience among young men and consequently may not precipitate intense feelings of shame. This is an area that may warrant further investigation in order to better understand what it is about these particular experiences of poverty that precipitate non-fatal suicidal behaviours among young men living under conditions of endemic poverty [20].

It is very significant that when we included measures of depression in our analysis of predictors of non-fatal suicidal behaviour, we found that symptoms of depression were by far the most significant predictor of non-fatal suicidal behaviour. In our data, symptoms of depression were a better predictor of non-fatal suicidal behaviour than any of the wide variety of poverty-related variables we considered. This finding is significant in the light of literature which contests the importance of psychiatric factors in the aetiology of suicide [15–18]. In spite of claims made in the critical suicidology literature (often without empirical evidence) about the primacy of socio-economic and cultural factors over psychiatric factors in the aetiology of suicide in LMICs [18], our data highlight the importance for policy makers to focus on psychiatric issues, like depression, in public health suicide prevention programmes, especially amongst those living in low-resource communities.

We know from five decades of epidemiological research that there are risk factors correlated with suicidal behaviour [2]; for example, being homeless, identifying as gay, being male, having a psychiatric illness, being poor, and having access to lethal means of self-harm [1]. In this study, we focused on a group who are all considered to be at high risk of suicide by virtue of the fact that they are poor Black African men who experience prejudice and face few opportunities to fulfil their male roles [1–3, 7, 9, 40]. Our data show clearly that among such a high-risk group, being fired, experiencing symptoms of depression and food insecurity are strongly associated with an increased risk of non-fatal suicidal behaviour. This finding supports the assumption that interventions to reduce the morbidity and mortality associated with non-fatal suicidal behaviour in this high-risk group of young men living under conditions of poverty need to be focused on proximal factors, such as promoting access to psychiatric care to reduce depressive symptoms, food security, re-employment and job security. Future research assessing the pathways between these particular experiences of poverty, symptoms of depression, and non-fatal suicidal behaviour will help identify the causal determinants of non-fatal suicidal behaviour in low resource contexts, helping provide more specific targets for suicide prevention interventions.

### Limitations

Data for this study were collected from two low-resource peri-urban communities in the Western Cape province of SA. As such, it is not clear how representative the findings are of other low-resource communities, particularly those in rural areas. A further limitation of this study is the inclusion of suicidal ideation and suicidal behaviour within the definition we used of non-fatal suicidal behaviour. It is possible that there are different risk factors for suicidal ideation and suicidal behaviour, and that these two phenomena should be investigated separately. This would, however, require further studies with very large sample sizes, as the base rate of suicidal behaviour is extremely low, making it difficult to yield enough statistical power to investigate how suicidal behaviour is influenced by the interaction between a wide variety of potential independent variables.

The meaning attributed to suicidal behaviour and the language used to describe these phenomena is shaped by cultural and contextual factors [41]. The language used in the C-SSRS which we utilised to assess non-fatal suicidal behaviour in this study was developed by researchers in the USA. Consequently, the instrument may have failed to capture cultural nuances in the descriptions of suicidal ideation and non-fatal suicidal behaviour.

While this study considered a wide range of poverty-related measures, it did not utilise a composite index of wealth or consider the value of household assets owned. It may be helpful for future studies in this area to incorporate a wealth index and not only consider measures of income as a proxy for poverty.

## Conclusion

Our data indicate that non-fatal suicidal behaviour among young black men living in low-resource communities is associated with particular dimensions of poverty, such as job loss and food insecurity, but not with other dimensions of poverty, such as prolonged unemployment and low levels of income. These findings support the view that socio-economic factors are among the proximal risk factors for non-fatal suicidal behaviour. However, our data also suggest that symptoms of depression are a better predictor of non-fatal suicidal behaviour than poverty-related factors among young men living under conditions of poverty in SA. These findings support the idea that, while economic variables may be associated with non-fatal suicidal behaviour, depressive symptoms confound the influence of poverty to precipitate non-fatal suicidal behaviours among young black men living in low-resource peri-urban SA communities. These findings call into question assertions by critical suicidologists that mental illness is not an important contributor to suicidal behaviour; symptoms of depression appear to be an important proximal risk factor which increase the risk of non-fatal suicidal behaviour among men living in poor communities in LMICs and should be the focus of suicide prevention interventions in these settings.

## Additional file

Additional file 1: Interview guide. (DOCX 34 kb)

## Abbreviations

WHO: World Health Organization
LMICs: Low- and middle-income countries
SA: South Africa
MDD: Major Depressive Disorder
HFIAS: Household Food Insecurity Access Scale
CES-D: Center for Epidemiologic Studies Depression Scale
C-SSRS: Columbia-Suicide Severity Rating Scale

## References

[1] World Health Organization (2014). Preventing suicide: A global imperative.

[2] Franklin JC, Ribeiro JD, Fox KR, Bentley KH, Kleiman EM, Huang X (2016). Risk factors for suicidal thoughts and behaviors: A meta-analysis of 50 years of research. Psychol Bull.

[3] Iemmi V, Bantjes J, Coast E, Channer K, Leone T, McDaid D (2016). Suicide and poverty in low-income and middle-income countries: a systematic review. Lancet Psychiatry.

[4] Bantjes J, Kagee A (2013). Epidemiology of suicide in South Africa: Setting an agenda for future research. S Afr J Psychol.

[5] Posner K, Brodsky B, Yershova K, Buchanan J, Mann J, Nock MK (2014). The classification of suicidal behavior. The Oxford handbook of Suicide and Self-injury.

[6] Silverman MM, Berman AL, Sanddal ND, O’Carroll PW, Joiner TE (2007). Rebuilding the tower of babel: a revised nomenclature for the study of suicide and suicidal behaviors part 1: background, rationale, and methodology. Suicide Life Threat Behav.

[7] Bertolote JM, Fleischmann A, De Leo D, Bolhari J, Botega N, De Silva D (2005). Suicide attempts, plans, and ideation in culturally diverse sites: the WHO SUPRE-MISS community survey. Psychol Med.

[8] Weissman MM, Bland RC, Canino GJ, Greenwald S, Hwu HG, Joyce PR (1999). Prevalence of suicide ideation and suicide attempts in nine countries. Psychol Med.

[9] Kessler RC, Borges G, Walters EE (1999). Prevalence of and risk factors for lifetime suicide attempts in the National Comorbidity Survey. Arch Gen Psychiatry.

[10] Posner K, Brown GK, Stanley B, Brent DA, Yershova KV, Oquendo MA (2011). The Columbia–Suicide Severity Rating Scale: initial validity and internal consistency findings from three multisite studies with adolescents and adults. Am J Psychiatry.

[11] Brent DA, Oquendo M, Birmaher B, Greenhill L, Kolko D, Stanley B (2002). Familial pathways to early-onset suicide attempt: risk for suicidal behavior in offspring of mood-disordered suicide attempters. Arch Gen Psychiatry.

[12] Fergusson DM, Horwood LJ, Ridder EM, Beautrais AL (2005). Suicidal behaviour in adolescence and subsequent mental health outcomes in young adulthood. Psychol Med.

[13] Wichstrom L (2000). Predictors of adolescent suicide attempts: A nationally representative longitudinal study of Norwegian adolescents. J Am Acad Child Adolesc Psychiatry.

[14] Brown GK, Beck AT, Steer RA, Grisham JR (2000). Risk factors for suicide in psychiatric outpatients: A 20-year prospective study. J Consult Clin Psychol.

[15] Marsh I (2010). Suicide: Foucault, history and truth.

[16] White J (2015). Shaking up suicidology. Soc Epistemol Rev Reply Collect.

[17] White J, Marsh I, Kral MJ, Morris J (2015). Critical suicidology: Transforming suicide research and prevention for the 21st century.

[18] Hjelmeland H, Dieserud G, Dyregrov K, Knizek BL, Rasmussen ML (2014). Suicide and mental disorders. Tidsskr Nor Laegeforen.

[19] McDaid D, Kennelly B, Wasserman D, Wasserman C (2009). An economic perspective on suicide across the five continents. Oxford textbook of suicidology and suicide prevention.

[20] Bantjes J, Iemmi V, Coast E, Channer K, Leone T, McDaid D (2016). Poverty and suicide research in low- and middle-income countries: systematic mapping of literature published in English and a proposed research agenda. Glob Ment Health.

[21] Hegerl U, Wittenburg L, Arensman E, Van Audenhove C, Coyne JC, McDaid D (2009). Optimizing suicide prevention programs and their implementation in Europe (OSPI Europe): an evidence-based multi-level approach. BMC Public Health.

[22] Rotheram-Borus MJ, Piacentini J, Miller S, Graae F, Castro-Blanco D (1994). Brief cognitive-behavioral treatment for adolescent suicide attempters and their families. J Am Acad Child Adolesc Psychiatry.

[23] Western Cape Government. Informal settlements status presentation (2013). 2013. https://www.westerncape.gov.za/files/hs-informal-settlements-presentation-27-november-2013.pdf. Accessed 29 June 2018.

[24] Western Cape Government. Socio-economic profile: City of Cape Town. 2015. https://www.westerncape.gov.za/assets/departments/treasury/Documents/Socio-economic-profiles/2016/municipality/00_city_of_cape_town_2015_sep-lg_profile_feb_2016.pdf. Accessed 29 June 2018.

[25] Kalichman SC, Simbayi LC, Vermaak R, Jooste S, Cain D (2008). HIV/AIDS risks among men and women who drink at informal alcohol serving establishments (Shebeens) in Cape Town, South Africa. Prev Sci.

[26] Knueppel D, Demment M, Kaiser L (2010). Validation of the household food insecurity access scale in rural Tanzania. Public Health Nutr.

[27] Swindale A, Bilinsky P (2006). Development of a universally applicable household food insecurity measurement tool: process, current status, and outstanding issues. J Nutr.

[28] Radloff LS (1977). The CES-D scale: A self-report depression scale for research in the general population. Appl Psychol Meas.

[29] Murphy JM, Tsuang MT, Tohen M (2002). Symptom scales and diagnostic schedules in adult psychiatry. Textbook in psychiatric epidemiology.

[30] Hamad R, Fernald LCH, Karlan DS, Zinman J (2008). Social and economic correlates of depressive symptoms and perceived stress in South African adults. J Epidemiol Community Health.

[31] Tomlinson M, Grimsrud AT, Stein DJ, Williams DR, Myer L (2009). The epidemiology of major depression in South Africa: results from the South African stress and health study. SAMJ: S Afr Med J.

[32] Peltzer K (2008). Social support and suicide risk among secondary school students in Cape Town, South Africa. Psychol Rep.

[33] Joe S, Stein DJ, Seedat S, Herman A, Williams DR (2008). Non-fatal suicidal behavior among South Africans. Soc Psychiatry Psychiatr Epidemiol.

[34] Lund C, Breen A, Flisher AJ, Kakuma R, Corrigall J, Joska JA (2010). Poverty and common mental disorders in low and middle income countries: A systematic review. Soc Sci Med.

[35] Lee S, Guo WJ, Tsang A, Mak AD, Wu J, Ng KL, Kwok K (2010). Evidence for the 2008 economic crisis exacerbating depression in Hong Kong. J Affect Disord.

[36] Meltzer H, Bebbington P, Brugha T, Jenkins R, McManus S, Stansfeld S (2010). Job insecurity, socio-economic circumstances and depression. Psychol Med.

[37] Lund C, Myer L, Stein DJ, Williams DR, Flisher AJ (2013). Mental illness and lost income among adult South Africans. Soc Psychiatry Psychiatr Epidemiol.

[38] Tarasuk VS, Beaton GH (1999). Household food insecurity and hunger among families using food banks. Can J Public Health.

[39] Wiklander M, Samuelsson M, Jokinen J, Nilsonne Å, Wilczek A, Rylander G (2012). Shame-proneness in attempted suicide patients. BMC Psychiatry.

[40] Niehaus Isak (2012). Gendered Endings: Narratives of Male and Female Suicides in the South African Lowveld. Culture, Medicine, and Psychiatry.

[41] Colucci E, Lester D. (Eds.). Suicide and culture: Understanding the context. Hogrefe Publishing. 2012.

