# Supplementary material for: Non-fatal suicidal behaviour, depression and poverty among young men living in low-resource communities in South Africa
Source: BMC Public Health. 2018 Oct 22;18:1195. doi: 10.1186/s12889-018-6104-3 (PMC6198370; doi:10.1186/s12889-018-6104-3)
Supplement: Supplementary file 1 — Interview guide. (DOCX 34 kb) [file 12889_2018_6104_MOESM1_ESM.docx]

**Interview Guide**

**Participant Demographics**

**Participant’s age**

What is your current age?

**Partnership status**

What is your current partnership status?

- Married
- Living together
- Causal relationship

**Participant education**

| What is the highest level of education you have completed? No schooling [0]  |
| --- |
| Grade 1 / Sub A [1]  |
| Grade 2 / Sub B [2]  |
| Grade 3 / Std 1 [3]  |
| Grade 4/ Std 2 [4]  |
| Grade 5 / Std 3 [5]  |
| Grade 6 / Std 4 [6]  |
| Grade 7 / Std 5 [7]  |
| Grade 8 / Std 6 [8]  |
| Grade 9 / Std 7 [9]  |
| Grade 10 / Std 8 [10]  |
| Grade 11/ Std 9 [11]  |
| Grade 12/ Matric [12]  |
| Post Matric Certificate / Diploma [13]  |
| Degree [14]  |
| Decline to answer [91]  |

**Income and employment**

**Work Type**

| Have you done any of the following types of work? Factory worker [1]  |
| --- |
| Farm worker [2]  |
| Sell drugs [3]  |
| Vendor [4]  |
| Gang member [5]  |
| Teacher [6]  |
| Driver [7]  |
| Shebeen work [8]  |
| Admin person [9]  |
| Health worker [10]  |
| Security [11]  |
| Worked in the mines [12]  |
| Builder [13]  |
| Janitor [14]  |
| Sex worker [15]  |
| Other [16]  |
| Never worked [17]  |

**Monthly income**

| How much money did you earn or receive last month? 0 to 499 Rand [1]  |
| --- |
| 500 to 1000 [2]  |
| 1001 to 2000 [3]  |
| 2001 to 5000 [4]  |
| 5001 to 8000 [5]  |
| 8000 and above [6]  |

**Assessment of income**

| Do you think that is a good income? Yes [1]  |
| --- |
| No [2]  |

**Monthly income 3 months ago**

| How much money did you earn or receive three months ago (anchor in time)? 0 to 499 Rand [1]  |
| --- |
| 500 to 1000 [2]  |
| 1001 to 2000 [3]  |
| 2001 to 5000 [4]  |
| 5001 to 8000 [5]  |
| 8000 and above [6]  |
| Dont know [99]  |

**Assessment of income 3 months**

| Do you think this is a good income? Yes [1]  |
| --- |
| No [2]  |

**Highest income ever**

What is most amount of money you have ever earned or received in a month?

**Income from parents**

Do you receive any money each month from your parents?

| Yes [1]  |
| --- |
| No [2]  |
|  |

**Income from parents amount**

How much money did you receive from your parents last month?

**Income from partner**

| Do you share any of your partner's income? Yes [1]  |
| --- |
| No [2]  |
| My partner has no income [3]  |

**Income from partner amount**

How much did you receive from your partner last month?

**Employment under 18**

| When you were under the age of 18 did your family let you work? Yes [1]  |
| --- |
| No [2]  |

**Longest Time Held Job**

| What is the longest amount of time that you have held a job? Never [1]  |
| --- |
| Less than one month [2]  |
| 2-3 months [3]  |
| 3-6 months [4]  |
| Six months - one year [5]  |
| More than one year [6]  |

**Jobs in the last year**

How many different jobs have you had in the last year (anchor in time)?

**Fired ever**

How many times in your life have you ever been fired from a job?

**Fired in last year**

How many times have you been fired in the last year?

**Number People Supporting Financially**

How many people are you supporting financially?

**Housing and living circumstances**

**Participant living circumstances**

How many people are living under the same roof as you (people who sleep in the household more than 2 nights each week)?

**Partner living with you**

| Is your partner one of these people? Yes [1] |
| --- |
| No [2] |
| I don't have a current partner [3] |

**Partner staying with you**

| How many nights in a week does your partner stay at your home? 2 [2] |
| --- |
| 3 [3] |
| 4 [4] |
| 5 [5] |
| 6 [6] |
| 7 [7] |

**Staying with partner**

| How many nights per week do you stay at your partner's house? 0 [0] |
| --- |
| 1 [1] |
| 2 [2] |
| 3 [3] |
| 4 [4] |
| 5 [5] |
| 6 [6] |
| 7 [7] |

**Participant living with their children**

| Do you live with any of your OWN children? Yes [1] |
| --- |
| No [2] |

**Number of children living with**

| How many of your OWN children are you currently living with? 1 [1] |
| --- |
| 2 [2] |
| 3 [3 or more] |

**Housing description**

| What best describes your housing? Formal brick structure on a separate yard [1] |
| --- |
| Informal dwelling / shack in backyard [2] |

**Water source**

| What is the main source of drinking water? Water in the home [1] |
| --- |
| Water on the premises [2] |
| Water from a community tap / public tank [3] |

**Household toilet**

| What toilet facilities does your household have? Flush toilet on the premises [1] |
| --- |
| Public [2] |
| Portable [3] |
| Bucket system [4] |
| Bush [5] |

**Electricity**

| Do you have electricity in your household? Yes [1] |
| --- |
| No [2] |

**Cooking fuel**

| What is your main source of fuel for cooking? Electricity [1] |
| --- |
| Paraffin [2] |
| Gas [3] |
| Coal [4] |
| Wood [5] |
| Other [95] |

**Food security**

**Participant Days of Hunger**

How many days in the passed WEEK have you gone hungry? (By this I mean days when you felt you didn't have enough to eat)

**Household Children Hunger**

How many days in the last WEEK have any of the children in your household gone hungry? (By this I mean days when you felt that your children needed to eat more.)

**Availability of food**

| Now I would like to ask you about food in your home in the last MONTH. For each of the following questions, consider what has happened in the past 30 days. Please answer whether this happened never, rarely (once or twice), sometimes (3-10 times), or often (more than 10 times) in the past 30 days? **(1) Household food supply:** Did you worry that your household would not have enough food? Never [1] |
| --- |
| Rarely [2] |
| Sometimes [3] |
| Often [4] |
| **(2) Kind of food:** Were you or any household member not able to eat the kinds of foods you preferred because of a lack of money? Never [1] |
| Rarely [2] |
| Sometimes [3] |
| Often [4] |
| **(3) Same food each day:** Did you or any household member eat just a few kinds of food day after day due to a lack of money? Never [1] |
| Rarely [2] |
| Sometimes [3] |
| Often [4] |
| **(4) Food not preferred:** Did you or any household member eat food that you preferred not to eat because of a lack of resources to obtain other types of food? Never [1] |
| Rarely [2] |
| Sometimes [3] |
| Often [4] |
| **(5) Smaller meal:** Did you or any household member eat a smaller meal than you felt you needed because there was not enough food? Never [1] |
| Rarely [2] |
| Sometimes [3] |
| Often [4] |
| **(6) Fewer meals in day:** Did you or any household member eat fewer meals in a day because there was not enough food? Never [1] |
| Rarely [2] |
| Sometimes [3] |
| Often [4] |
| **(7) Go to sleep hungry:** Did you or any household member go to sleep at night hungry because there was not enough food? Never [1] |
| Rarely [2] |
| Sometimes [3] |
| Often [4] |
| **(8) Whole day not eating:** Did you or any household member go a whole day without eating anything because there was not enough food? Never [1] |
| Rarely [2] |
| Sometimes [3] |
| Often [4] |
| **(9) No food at all:** Was there ever no food at all in your household because there were not resources to get more? Never [1] |
| Rarely [2] |
| Sometimes [3] |
| Often [4] |

**Symptoms of Depression**

Below are listed ways that describe how you might have felt or behaved during the past week. Please tell me if these statements describe the way you felt or behaved in the past week.

**Being Bothered**

| I was bothered by things that usually don't bother me. None of the time [0] |
| --- |
| A little of the time [1] |
| Occasionally [2] |
| Most of the time [3] |

**Poor Appetite**

| I did not feel like eating: my appetite was poor. None of the time [0] |
| --- |
| A little of the time [1] |
| Occasionally [2] |
| Most of the time [3] |

**Feeling the Blues**

| I felt that I could not shake off the blues even with help from my family and friends. None of the time [0] |
| --- |
| A little of the time [1] |
| Occasionally [2] |
| Most of the time [3] |

**Feeling Equal**

| I felt that I was just as good as other people. None of the time [0] |
| --- |
| A little of the time [1] |
| Occasionally [2] |
| Most of the time [3] |

**Lack of Focus**

| I had trouble keeping my mind on what I was doing. None of the time [0] |
| --- |
| A little of the time [1] |
| Occasionally [2] |
| Most of the time [3] |

**Feeling Depressed**

| I felt depressed. None of the time [0] |
| --- |
| A little of the time [1] |
| Occasionally [2] |
| Most of the time [3] |

**Effort**

| I felt that everything I did was an effort. None of the time [0] |
| --- |
| A little of the time [1] |
| Occasionally [2] |
| Most of the time [3] |

**Hope for Future**

| I felt hopeful about the future. None of the time [0] |
| --- |
| A little of the time [1] |
| Occasionally [2] |
| Most of the time [3] |

**Life a Failure**

| I thought that my life had been a failure. None of the time [0] |
| --- |
| A little of the time [1] |
| Occasionally [2] |
| Most of the time [3] |

**Fear**

| I was fearful. None of the time [0] |
| --- |
| A little of the time [1] |
| Occasionally [2] |
| Most of the time [3] |

**Restless Sleep**

| My sleep was restless. None of the time [0] |
| --- |
| A little of the time [1] |
| Occasionally [2] |
| Most of the time [3] |

**Happy**

| I was happy. None of the time [0] |
| --- |
| A little of the time [1] |
| Occasionally [2] |
| Most of the time [3] |

**Quieter Than Usual**

| I talked less than usual. None of the time [0] |
| --- |
| A little of the time [1] |
| Occasionally [2] |
| Most of the time [3] |

**Lonely**

| I felt lonely. None of the time [0] |
| --- |
| A little of the time [1] |
| Occasionally [2] |
| Most of the time [3] |

**Unfriendly People**

| I felt that people were unfriendly. None of the time [0] |
| --- |
| A little of the time [1] |
| Occasionally [2] |
| Most of the time [3] |

**Enjoyment of Life**

| I enjoyed life. None of the time [0] |
| --- |
| A little of the time [1] |
| Occasionally [2] |
| Most of the time [3] |

**Crying**

| I had crying spells. None of the time [0] |
| --- |
| A little of the time [1] |
| Occasionally [2] |
| Most of the time [3] |

**Sadness**

| I felt sad. None of the time [0] |
| --- |
| A little of the time [1] |
| Occasionally [2] |
| Most of the time [3] |

**People Dislike Me**

| I felt that people dislike me. None of the time [0] |
| --- |
| A little of the time [1] |
| Occasionally [2] |
| Most of the time [3] |

**Motivation**

| I could not get 'going' None of the time [0] |
| --- |
| A little of the time [1] |
| Occasionally [2] |
| Most of the time [3] |

**Non-fatal suicidal behaviour**

All the questions that I am going to ask you now refer to how you felt or what you have done in the LAST SIX MONTH. So in the last month did you:

**Deliberate self-harm (in the last six months)**

| Have you deliberately hurt yourself in any way in the last six months? Yes [1] |
| --- |
| No [2] |

**Deliberate self-harm**

| In the last month have you deliberately harmed yourself in any way? Yes [1] |
| --- |
| No [2] |

**Passive suicidal ideation (in the last six months)**

| Have you had thoughts of death, wished to be dead, or dreamed of your own death in the last six months? Yes [1] |
| --- |
| No [2] |

**Passive suicidal ideation**

| Have you had thoughts of deaths, wished to be dead or dreamed of your own death in the last month? Yes [1] |
| --- |
| No [2] |

**Active suicidal ideation (in the last six months)**

| In the last six months have you had thoughts about killing yourself? Yes [1] |
| --- |
| No [2] |

**Active suicidal ideation**

| Have you had thoughts of killing yourself in the last month? Yes [1] |
| --- |
| No [2] |

**Suicidal plan (in the last six months)**

| In the last six months have you planned to kill yourself? Yes [1] |
| --- |
| No [2] |

**Suicide plan**

| Have you planned to kill yourself in the last month? Yes [1] |
| --- |
| No [2] |

**Suicidal attempt (in the last six months)**

| In the last six months have you tried to kill yourself? Yes [1] |
| --- |
| No [2] |

**Suicide attempt**

| Have you tried to kill yourself in the last month? Yes [1] |
| --- |
| No [2] |
